# Qiliqiangxin improves cardiac function and attenuates cardiac remodelling in doxorubicin-induced heart failure rats

**DOI:** 10.1080/13880209.2020.1761403

**Published:** 2020-05-19

**Authors:** Xutao Sun, Guozhen Chen, Ying Xie, Deyou Jiang, Jieru Han, Fei Chen, Yunjia Song

**Affiliations:** a School of Basic Medical Sciences, Heilongjiang University of Chinese Medicine, Harbin, Heilongjiang, P. R. China; b Department of Pediatrics, The Affiliated Yantai Yuhuangding Hospital of Qingdao University, Yantai, Shandong, P. R. China; c Peking University First Hospital, Beijing, P. R. China

**Keywords:** Chronic heart failure, miRNAs, TGF-β1/Smad3, TGF-β3/Smad7

## Abstract

**Context:**

Therapeutic doxorubicin administration is restricted as this anticancer drug may be cardiotoxic. The traditional Chinese medicine qiliqiangxin has been approved for clinical treatment of chronic heart failure.

**Objective:**

To explore the protective effects and molecular mechanisms of qiliqiangxin on doxorubicin-induced congestive heart failure (CHF) in rats.

**Materials and methods:**

A CHF rat model was established via intraperitoneal DOX injections (2.5 mg/kg/week) for 6 weeks. The rats were randomly assigned to control, CHF, CHF + QL (1.0 g/kg/d), or captopril (3.8 mg/kg/d) treatment groups (*n* = 10) for 4 weeks. MicroRNA sequencing elucidated the molecular mechanisms of qiliqiangxin on doxorubicin-induced CHF in rats.

**Results:**

Unlike in the CHF group, QL significantly reduced Bax:Bcl-2 (2.05 ± 0.23 vs. 0.94 ± 0.09, *p* < 0.05) and the levels of collagen I (0.19 ± 0.02 vs. 0.15 ± 0.01, *p* < 0.05), collagen III (0.19 ± 0.02 vs. 0.14 ± 0.02, *p* < 0.05), TGF-β1 (5.28 ± 0.89 vs. 2.47 ± 0.51, *p* < 0.05), Smad3 (1.23 ± 0.12 vs. 0.78 ± 0.09, *p* < 0.05), MMP-2 (0.89 ± 0.01 vs. 0.53 ± 0.05, *p* < 0.05), and TIMP-2 (0.24 ± 0.03 vs. 0.44 ± 0.03, *p* < 0.05). QL also upregulated TGF-β3 (0.65 ± 0.06 vs. 0.96 ± 0.10, *p* < 0.05) and Smad7 (0.09 ± 0.01 vs. 0.19 ± 0.023, *p* < 0.05). Moreover, Smad3 was a target of miR-345-3p.

**Discussion and Conclusions:**

The beneficial effects of QL on DOX-induced CHF in rats are mediated by reduction in myocardial fibrosis, promotion of TGF-β3/Smad7, and inhibition of TGF-β1/Smad3. QL may also modulate specific miRNAs. These results provide evidence that QL might be an effective treatment for DOX-induced CHF.

## Introduction

Doxorubicin (DOX) is an anthracycline antibiotic. It has been widely used in cancer chemotherapy as it intercalates with DNA, stabilises topoisomerase II (Top2), and inhibits DNA replication. However, its clinical application is restricted as it may be cardiotoxic and cause heart failure (Mazevet et al. 2013). DOX-induced cardiotoxicity is irreversible and eventually progresses to congestive heart failure (CHF), left ventricular remodelling, and dilated cardiomyopathy (Lipshultz et al. 2012). DOX-induced cardiac injury is characterised by mitochondrial oxidative stress, cardiomyocyte apoptosis, inflammatory reactions, interstitial fibrosis, cardiac remodelling, and eventual cardiac dysfunction (Shabalala et al. 2017; Zhang et al. 2017). Of these, cardiomyocyte inflammation and fibrosis are ordinary pathological mechanisms of myocardial remodelling in DOX-induced CHF (Liu et al. 2016; Chakraborti et al. 2018). The TGF-β superfamily includes the subtypes TGF-β1, TGF-β2, and TGF-β3. Each of these has its own distinct biological functions (Chen et al. 2018). TGF-β1 is thought to be a key proliferative and fibrogenic factor in cardiac, renal, hepatic, and pulmonary fibrosis. TGF-β3 is antagonistic to TGF-β1 (Katz et al. 2016; Eser and Jänne 2018). TGF-β1 also participates in cardiac remodelling. There is evidence that TGF-β1 is upregulated when TGF-β3 is downregulated and their subcellular distributions differentiate as left ventricular hypertrophy develops in rats (Li and Brooks 1997). Smad2 and Smad3 facilitate TGF-β1-mediated tissue fibrosis whereas Smad7 is a reverse-feedback regulator of the TGF-β1/Smad pathway and suppresses TGF-β1-induced fibrosis (Deng et al. 2017). Moreover, nuclear factor κB (NF-κB) plays significant roles in inflammation and has important influences on cardiac remodelling and cardiac failure (Deten et al. 2002; Frantz et al. 2003; Christia and Frangogiannis 2013). Inactivated NF-κB dimers combine to inhibit kappa B (IκB) proteins. Degraded IκB is then released and induces NF-κB which then causes the transcription of matrix metalloproteinases (MMPs), tissue inhibitor of matrix metalloproteinases (TIMPs), MCP-1, TNF-α, and IL-6. It also stimulates myocardial inflammation and fibrotic responses that lead to cardiac remodelling. Numerous animal models have been used to show that NF-κB suppression may attenuate cardiac fibrosis (Xie et al. 2004; Nam 2006; Timmers et al. 2009).

MicroRNAs (miRNAs) are short genome-encoded, internally transcribed, non-translated RNAs ∼18 to 23 nucleotides long. They were first discovered in plants (Lee et al. 1993). They strongly affect post-transcriptional target gene regulation by destabilising mRNA and suppressing its translation (Filipowicz et al. 2008). MiRNAs may influence various pathophysiological processes such as biosome growth, immunity, and carcinomatosis (Cullen 2006; Marson et al. 2008; Croce 2009). MiRNAs play vital roles in cardiac development and pathophysiology (Latronico and Condorelli 2009). These properties make miRNAs attractive biomarkers and therapeutic targets for the treatment of cardiovascular diseases.

Qiliqiangxin (QL) is a standardised traditional Chinese herbal remedy that has been shown to have good therapeutic efficacy against CHF as it improves cardiac function, attenuates myocardial remodelling, alleviates cardiac fibrosis, inhibits apoptosis, induces myocardial regeneration, and enhances angiogenesis (Zou et al. 2012; Tang and Huang 2013). However, it is unknown whether or how QL protects cardiomyocytes from DOX-induced CHF. Therefore, in the present study, a DOX-induced CHF model was used to assess the relative efficacy of QL at improving cardiac function and attenuating myocardial remodelling and to investigate its relevant molecular mechanisms.

## Materials and methods

### Therapeutic materials

QL capsules were provided by Yiling Pharmaceutical Corporation (Shijiazhuang, China). The QL powder consisted of *Panax ginseng* C. A. Mey. (Araliaceae)*, Astragalus membranaceus* Bge. (Fabaceae), *Salvia miltiorrhiza* Bge.(Lamiaceae), *Descurainia sophia* L. (Brassicaceae), *Aconitum carmichaeli* Debx. (Ranunculaceae), *Alisma orientalis* Sam. (Alismataceae), *Carthamus tinctorius* L. (Asteraceae), *Acanthopanax gracilistylus* W. W. Smith. (Araliaceae)*, Polygonatum odoratum* Mill. (Asparagaceae), *Cinnamomum cassia* L. (Lauraceae), and *Citrus aurantium* L. (Rutaceae). It was dissolved in sterile water to a concentration of 0.1 g/mL. Doxorubicin (DOX; Batch No. 020150703; Shanxi Pude Pharmaceutical Co. Ltd., Datong, Shanxi, China) and the angiotensin-converting enzyme (ACE) inhibitor captopril (Batch No. 1511221; Suicheng Pharmaceutical Co. Ltd., Zhengzhou, Henan, China) were separately dissolved in sterile saline solution.

### Animals

Male Wistar rats, each weighing 180–200 g, were purchased from Beijing Huafukang Biotechnology Co. Ltd., Beijing, China (animal licence No. SYXK (Jing) 2014-0010). They were maintained on standard food and water at 25 ± 1 °C, a 12 h light/dark cycle, and 50 ± 5% RH. All animal testing procedures were conducted in accordance with the Ethics Committee Guidelines on the Care and Use of Laboratory Animals (NIH Publication No. 85-23, revised 1996). Thirty-three rats were injected with 2.5 mg/kg/week DOX for 6 weeks to induce CHF model, during which three rats died. After the final injection, rat cardiac function was measured by echocardiography. The rats were randomly assigned to the control, CHF, CHF + QL (1.0 g/kg/d), or captopril (3.8 mg/kg/d) group (n = 10 per group) and underwent intragastric treatment for another 4 weeks, during which three rats died in the CHF group, and none rat died in the CHF + QL and captopril group.

### Echocardiography

After anaesthesia, the rats were fixed on fadeaway posts to enable access to the long axis of their left ventricles. They were fitted with 7 to 12-MHz phased-array transducers (Vivid7; General Electric Co., Boston, MA\). The left ventricular diastolic and systolic internal diameters (LVIDd/LVIDs) and the ejection fraction (EF) and shortening fraction (FS) were measured. The averages of ≥3 measurements per rat were calculated.

### Hemodynamics

After the echocardiographic recordings, the animals were maintained under anaesthesia and a tip-transducer tube (1 mm) was inserted into the right arteria carotis to record the following hemodynamic parameters: left ventricular end-diastolic pressure (LVEDP), left ventricular systolic pressure (LVSP), and maximum pressure positive and negative velocity (± dp/dt). Data were analysed by PowerLab software (ADInstruments, Dunedin, NZ). The rats were then euthanized by anaesthetic overdose. Their hearts were excised, sectioned at the midpoint of the long axis of the left ventricle, and preserved for histologic analysis.

### Histopathology

#### Hematoxylin-eosin (HE) staining

Myocardium specimens were washed with phosphate-buffered saline (PBS), immobilised in 4% (v/v) triformol overnight, dehydrated, rendered transparent, and embedded with paraffin. The tissues were sectioned by microtome and the slices were stained with hematoxylin-eosin (HE). Histopathological changes in the myocardial tissues were observed under an optical microscope.

### Immunohistochemistry

Immunohistochemical (IHC) staining was used to assess the protein levels of Bax, Bcl-2, TGF-β1, TGF-β3, Col-I, Col-III, MMP-2, and TIMP-2. The tissue specimens were processed as described above. Paraffin-embedded myocardium was sliced to 4 µm thickness and mounted on slides. The slices were deparaffinized, dehydrated, incubated in 3% (v/v) H_2_O_2_ at room temperature for 10 min, incubated with pepsin at 37 °C for 30 min to restore antigen activity, and blocked with 5% (v/v) goat serum at room temperature for 30 min. The sections were then incubated with primary antibodies (Bax, 1:100; Bcl-2, 1:100; TGF-β3, 1:100; TGF-β1, 1:100; Col-I, 1:100; Col-III, 1:100; MMP-2, 1:200; and TIMP-2, 1:200) overnight at 4 °C. The next day, the slides were washed three-times in PBS for 5 min per rinse. Biotin-labeled secondary antibody was incubated at 37 °C for 1 h. The tissue slices were washed three-times with PBS for 5 min per rinse, stained with HE, rinsed, and viewed at ×400 under an optical microscope (Motie3000).

### Enzyme-linked immunosorbent assay (ELISA)

ELISA was conducted to determine cardiac MMP-2 and TIMP-2 expression using commercially available rat MMP-2 and TIMP-2 kits (Biotop Oy, Turku, Finland) in accordance with the manufacturer’s instructions. Monoclonal antibodies specific for rat MMP-2 and TIMP-2 were placed in the ELISA well. Standards and samples were added to the microplates, polyclonal antibodies were conjugated to horseradish peroxidase, and the plates were incubated. The substrates and the final solutions were added in succession to the microplates. The optical density of each well was detected by ELISA at 450 nm and the MMP-2 and TIMP-2 values were interpolated from standard curves.

### Quantitative real-time polymerase chain reaction (qRT-PCR)

Heart tissue samples were pre-treated as described above and total RNA was extracted from them with TRIzol^®^ reagent (Invitrogen, Carlsbad, CA). The RNA was reverse-transcribed to cDNAs using an iScript™ cDNA synthesis kit (Bio-Rad Laboratories, Hercules, CA) according to the manufacturer’s specifications. The qRT-PCR was then performed with the CFX Connect real-time PCR system (Bio-Rad Laboratories, Hercules, CA) and a SYBR-Green Supermix kit (Bio-Rad Laboratories, Hercules, CA). The forward- and reverse primer sequences used in this study are listed in Table 1. The cycle threshold and 2^−ΔΔct^ methods were used for the relative quantification of mRNA expression.

**Table 1.** Primers designed for qRT-PCR.

Gene	Primer
*TGF-β1*	F: AGAAGTCACCCGCGTGCTA
	R: AGAAGTCACCCGCGTGCTA
*TGF-β3*	F: AGAAGTCACCCGCGTGCTA
	R: TCATCTTCGTTGTCCACTCCT
*Smad3*	F: ACAGCATGGACGCAGGTTCT
	R: TCACTGAGGCACTCCGCAAA
*Smad7*	F: TGGATGGCGTGTGGGTTTA
	R: TGGCGGACTTGATGAAGATG
*miR-345-3p*	RT: CTCAACTGGTGTCGTGGAGTCGGCAATTCAGTTGAGGGACTTGA
	F: GCCGAGAGGGGTCTGGAGA
	R: CTCAACTGGTGTCGTGGA
*miR-328a-3p*	RT: CTCAACTGGTGTCGTGGAGTCGGCAATTCAGTTGAG ACCGGGAG
	F: GCCGAGTGCCTTCCCGTCT
	R: CTCAACTGGTGTCGTGGA
*miR-106b-3p*	RT: CTCAACTGGTGTCGTGGAGTCGGCAATTCAGTTGAGGCGTGACA
	F: GCCGAGGGGTACTTGCTGC
	R: CTCAACTGGTGTCGTGGA

### Western blot

After the treatments were administered, all animals were euthanized and their myocardia were rapidly excised and preserved in liquid nitrogen. Anti-Smad3 (phosphor C25A9) (1:500, Cell Signalling Technology, Inc., Danvers, MA) and anti-Smad7 (1:500, Cell Signalling Technology, Inc., Danvers, MA) were added to the tissue samples. Horseradish peroxidase-labeled secondary antibodies (Sigma-Aldrich Corp., St. Louis, MO) were diluted to 1:2000 and added to the tissue samples. The proteins were separated by 12% (w/v) SDS-PAGE, mounted on nylon membrane, and incubated with primary antibodies at 4 °C overnight. The membranes were visualised with a FluorChem MultiFluor System (ProteinSimple, San Jose, CA).

### miRNA-seq and bioinformatics

Total RNA was extracted with the RNeasy mini kit (Cat No. 74104; Qiagen, Hilden, Germany) from the left ventricular myocardial tissue of five untreated DOX-induced CHF rats and five DOX-induced CHF rats treated with QL. RNA concentration and purity were determined by spectrophotometry (NanoDrop 2000^TM^; Thermo Fisher Scientific, Wilmington, DE). The miRNA-seq tests were run in triplicate using the Affymetrix GeneChip miRNA 4.0 Array (Thermo Fisher Scientific, Waltham, MA). Differentially expressing miRNAs (DEmiRs) between the untreated CHF group and the CHF group treated with QL were analysed with the Gene-Cloud of Biotechnology Information (GCBI) platform. DEmiRs targets were predicted by target scan and miRanda. A volcano plot was constructed in R v. 5.50 (Mathsoft Engineering and Education, Inc., Cambridge, MA). Hierarchical clustering was executed with Cluster v. 3.0 (http://bonsai.hgc.jp/∼mdehoon/software/cluster/software.htm).

### miRNA target luciferase reporter assay

To detect luciferase reporter vectors, the 3′-UTR of rat Smad3 was cloned downstream to the Renilla psiCHECK2 vector (Promega, Madison, WI) and named wtSmad3 3′-UTR. To construct the mutant Smad3 reporter, the seed region of the Smad3 3′-UTR was mutated to eliminate all supplements with nucleotides 2-7 of miR-345-3p, miR-328a-3p, and miR-106b-3p and named MUTSmad3 3′-UTR. The carriers were co-transfected into HEK 293 cells with miR-345-3p, miR-328a-3p, and miR-106b-3p mimics and the luciferase activity was detected.

### HEK 293 cell culture and transfection

HEK 293 (CRL-1573, ATCC) cells were cultured in minimum essential medium (MEM; Sigma-Aldrich Corp., St. Louis, MO) supplemented with 10% (v/v) heat-inactivated foetal bovine serum (FBS; Invitrogen, Carlsbad, CA). Cells (76,105 per well) were co-transfected with the reporter vectors (70 ng per well) and the integrated mmu-miR-345-3p, miR-328a-3p, and miR-106b-3p mimics (40 nM or 80 nM; Dharmacon, Lafayette, CO) using Lipofectamine 2000^TM^ (Invitrogen, Carlsbad, CA).

### Statistical analysis

Data are presented as means ± standard error (SE). Differences in treatment means among groups were calculated by one-way ANOVA in SPSS software (IBM Corp., Armonk, NY). *p* < 0.05 was considered statistically significant.

## Results

### QL alleviated DOX-induced cardiac dysfunction

**Figure 1.**
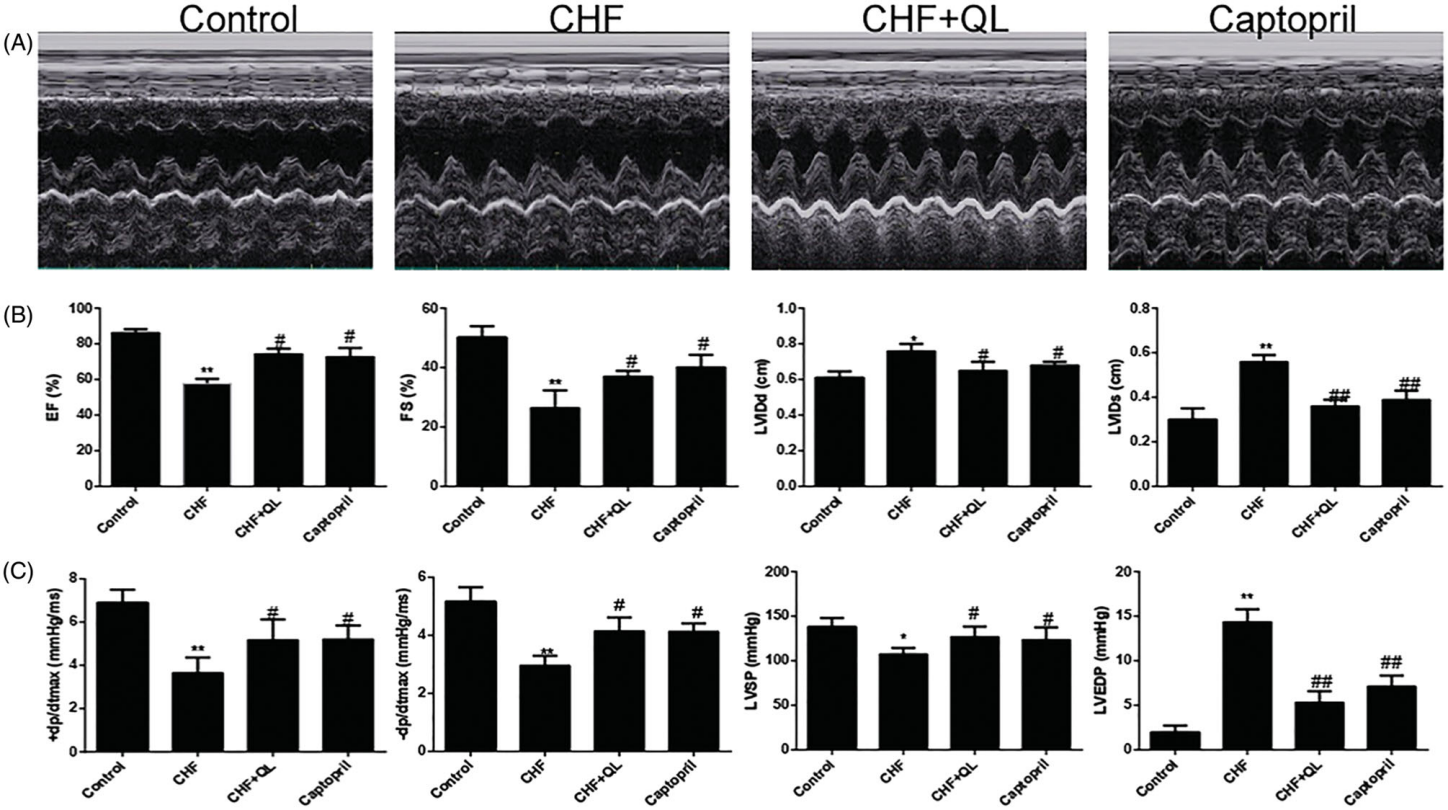
QL restored cardiac function in DOX-induced CHF. (A) M-mode echocardiographic images of each treatment group. (B) Cardiac function was determined by EF, FS, LVIDd and LVIDs and compared among the different groups by echocardiography. (C) Comparison of the hemodynamic parameters LVSP, LVEDP, +dp/dtmax and − dp/dtmax among different groups after QL treatment. *p < 0.05, **p < 0.01 vs. control group. #*p* < 0.05, ##*p* < 0.01 vs. CHF group.

Echocardiography and hemodynamics studies revealed significant impairment in LV function after DOX infusion, indicated by increased LVIDd, LVIDs, and LVEDP and decreased EF, FS, LVSP, +dp/dtmax, and − dp/dtmax compared to the values observed in the unchallenged control. QL and captopril substantially improved myocardial function, as indicated by increased EF, FS, LVSP, +dp/dtmax, and − dp/dtmax and decreased LVIDd, LVIDs, and LVEDP relative to values in the CHF model group (Figure 1).

### QL improved myocardium ultrastructure in DOX-induced CHF rats

**Figure 2.**
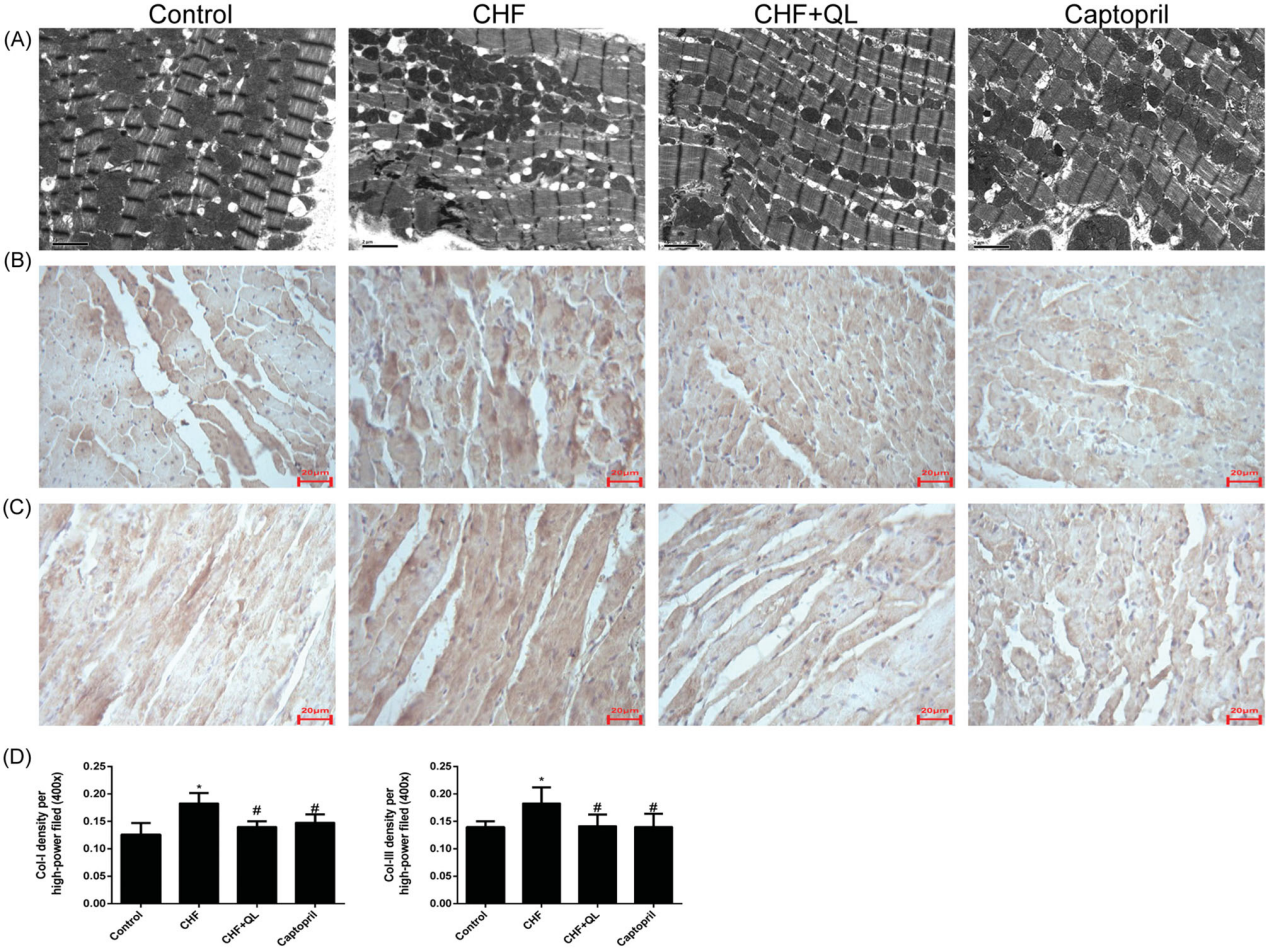
QL improved myocardium ultrastructure and reduced fibrosis. (A) TEM images of left ventricles of rats. (B, C) IHC disclosed that QL markedly downregulated collagens I and III. (D) Collagens I and III positive integral optical density. *p < 0.05, **p < 0.01 vs. control group. #*p* < 0.05, ##*p* < 0.01 vs. CHF group.

Figure 2(A) shows transmission electron microscope (TEM) images of the myocardial ultrastructure of DOX-induced CHF rats. Their sarcomeres were destroyed and their mitochondria were swollen and damaged. In contrast, the QL and captopril treatments attenuated or mitigated these toxic effects.

### QL inhibited fibrosis in rats with DOX-induced CHF

Representative immunohistochemical images revealed that the levels of collagens I and III were downregulated in the left ventricles of rats treated with QL and captopril compared to those in the CHF model (Figures 2(B,C)). Therefore, QL suppressed fibrosis in rats with DOX-induced CHF.

### QL attenuated cardiomyocyte apoptosis and inflammation

**Figure 3.**
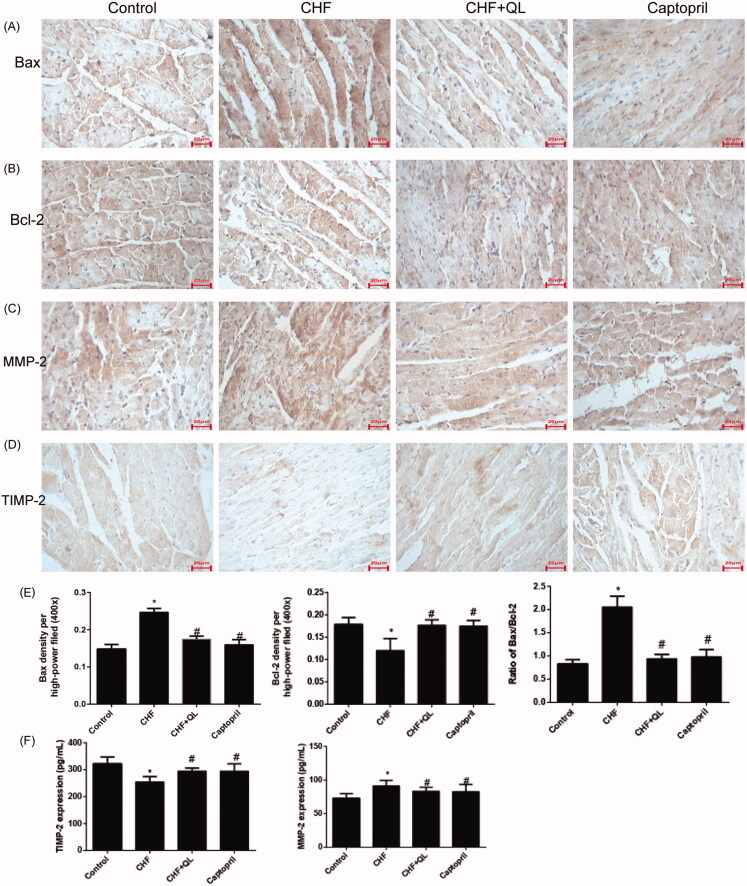
QL suppressed apoptosis and inflammation in DOX-induced CHF rats. (A, B) IHC images showing that QL downregulated Bax and upregulated Bcl-2. (C, D) ICH images showing that QL treatment downregulated MMP-2 and TIMP-2. (E) Analysis of Bax and Bcl-2 positive integral optical density. (F) ELISA demonstrated that QL substantially downregulated MMP-2 and TIMP-2. *p < 0.05, **p < 0.01 vs. control group. #*p* < 0.05, ##*p* < 0.01 vs. CHF group.

DOX application increased cardiomyocyte apoptosis. Immunohistochemistry indicated that Bax was markedly upregulated but Bcl-2 was markedly downregulated in the CHF model. QL and captopril administration upregulated Bcl-2 and downregulated Bax, thereby lowering the elevated Bax/Bcl-2 ratio characteristic of the CHF model (Figure 3(A,B)). The immunohistochemistry and ELISA data, shown in Figures 3(C,D), indicate that the inflammatory proteins MMPs and TIMPs were substantially upregulated in the CHF group compared to the levels in the control group and were markedly reduced in the QL group. Thus, QL suppressed apoptosis and inflammation in rats with DOX-induced CHF.

### QL promoted cardiac TGF-β3/Smad7 and inhibited cardiac TGF-β1/Smad3 signalling pathways in rats with DOX-induced CHF

**Figure 4.**
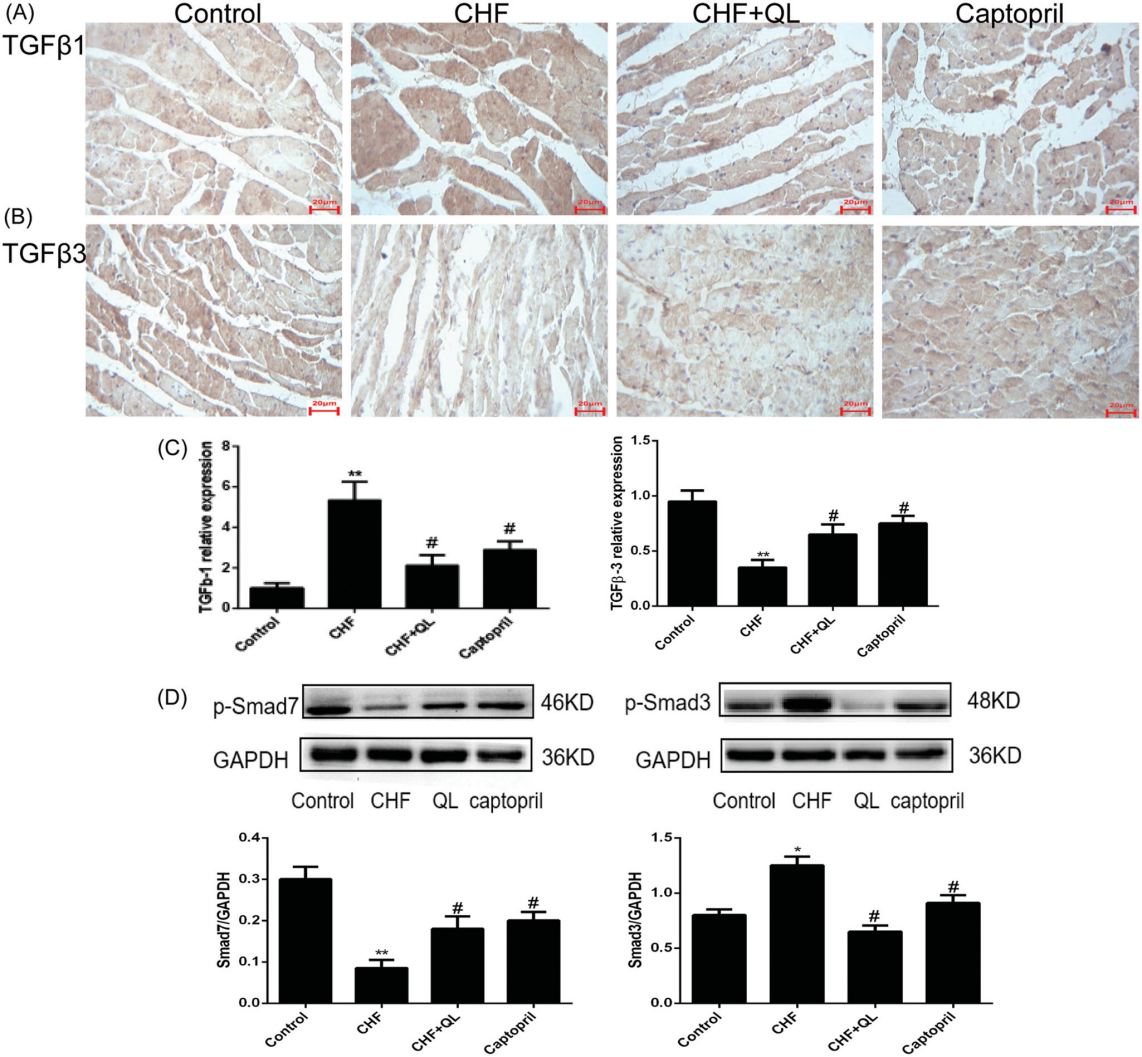
QL promoted the cardiac TGF-β3/signalling pathway Smad7 and inhibited the TGF-β1/Smad3 signalling pathway. (A–C) IHC and qRT-PCR indicated that the protein expression levels of TGF-β3 and Smad7 were increased while those of TGF-β1 and Smad3 were decreased in response to QL and captopril. (D) WB of p-Smad7 and p-Smad3. *p < 0.05, **p < 0.01 vs. control group. #*p* < 0.05, ##*p* < 0.01 vs. CHF group.

Previous studies have shown that TGF-β1 contributes to numerous pathophysiological fibrotic courses, whereas TGF-β3 has antifibrotic capabilities particularly in wound healing and in pulmonary and hepatic fibrosis. Therefore, we endeavoured to elucidate the functions of TGF-β1 and TGF-β3 in DOX-induced myocardial fibrosis. qRT-PCR and immunohistochemical analyses revealed that the mRNA and protein levels of TGF-β3 and Smad7 were considerably reduced, whereas those of TGF-β1 and Smad3 were substantially enhanced in the CHF model compared to those of the control. In contrast, QL and captopril administration both upregulated TGF-β3 and Smad7 and downregulated TGF-β1 and Smad3 levels (Figure 4). Taken together, these data suggest that TGF-β3 may regulate myocardial fibrosis and is antagonistic to TGF-β1.

### miRNA profiling identified and predicted miRNA gene targets

**Figure 5.**
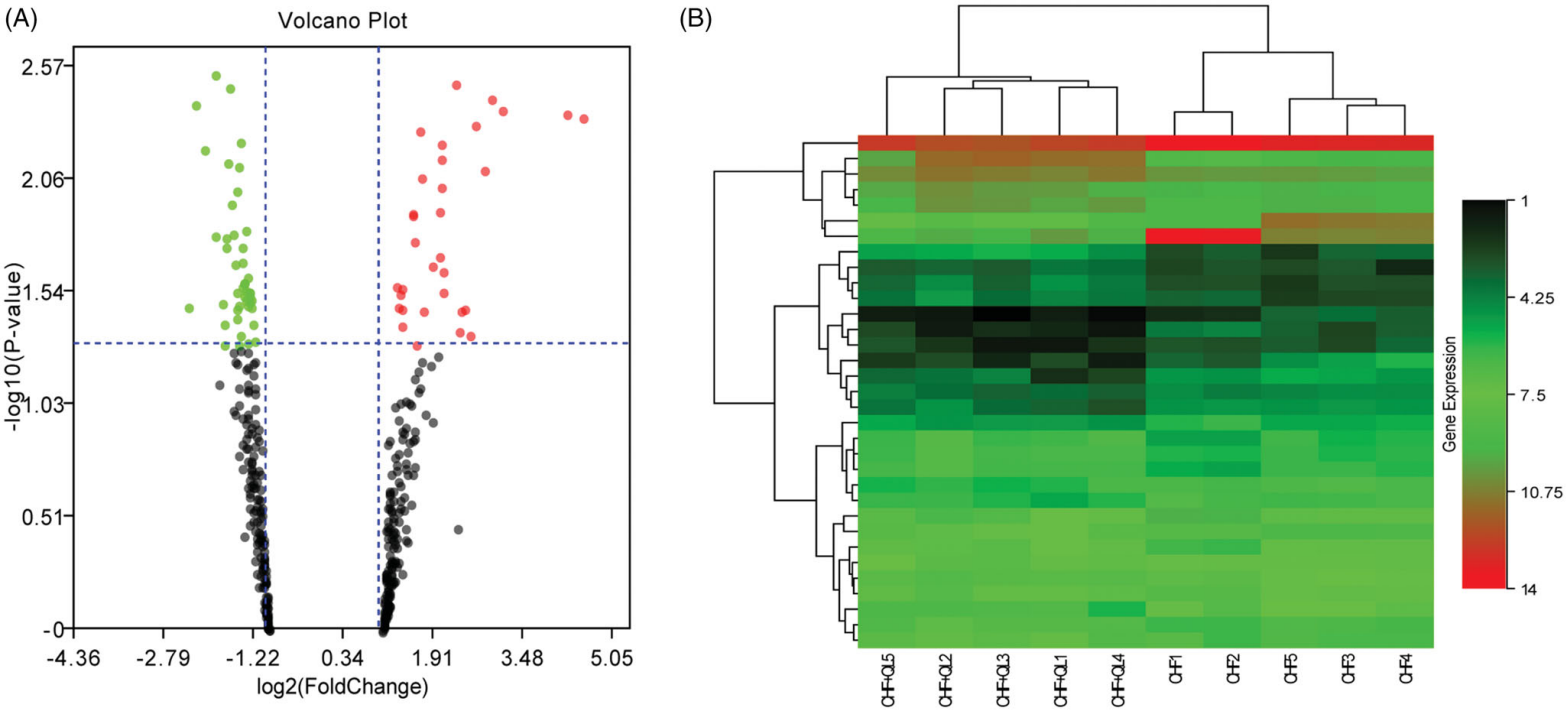
miRNA-seq data corresponding to DEmiRNAs between CHF model and QL groups. (A) Volcano plots of transformed log2 fold changes against *p*-values (−log10) corresponding to transcripts per million reads under both conditions examined. Green and red dots represent differentially expressed miRNAs. Black dots represent miRNAs not differentially expressed. (B) Hierarchical cluster of DEmiRNAs in the CHF vs. QL groups. Average DEmiRNA signals in each group were clustered with a Euclidean distance function. MiRNAs exhibiting similar expression patterns were clustered together (*n* = 5 per group). MiRNA-seq: miRNA-sequencing; DEmiRNAs: differentially expressed miRNAs.

**Figure 6.**
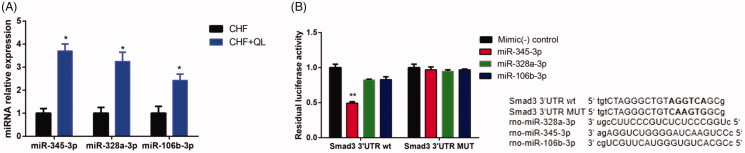
Analyses of miR-345-3p, miR-328a-3p, and miR-106b-3p. (A) Expression levels of three miRNAs quantified by qRT-PCR. (B) Analysis of luciferase activity. Sequence alignment of miR-345-3p, miR-328a-3p, miR-106b-3p, and their target sites in the 3′ UTR of Smad3 wt and MUT. Normalized luciferase activities of three miRNA mimics were set to relative luciferase activity confirmed in a *C. elegans* negative control mimic. *p < 0.05, **p < 0.01 vs. CHF/control mimic.

**Figure 7.**
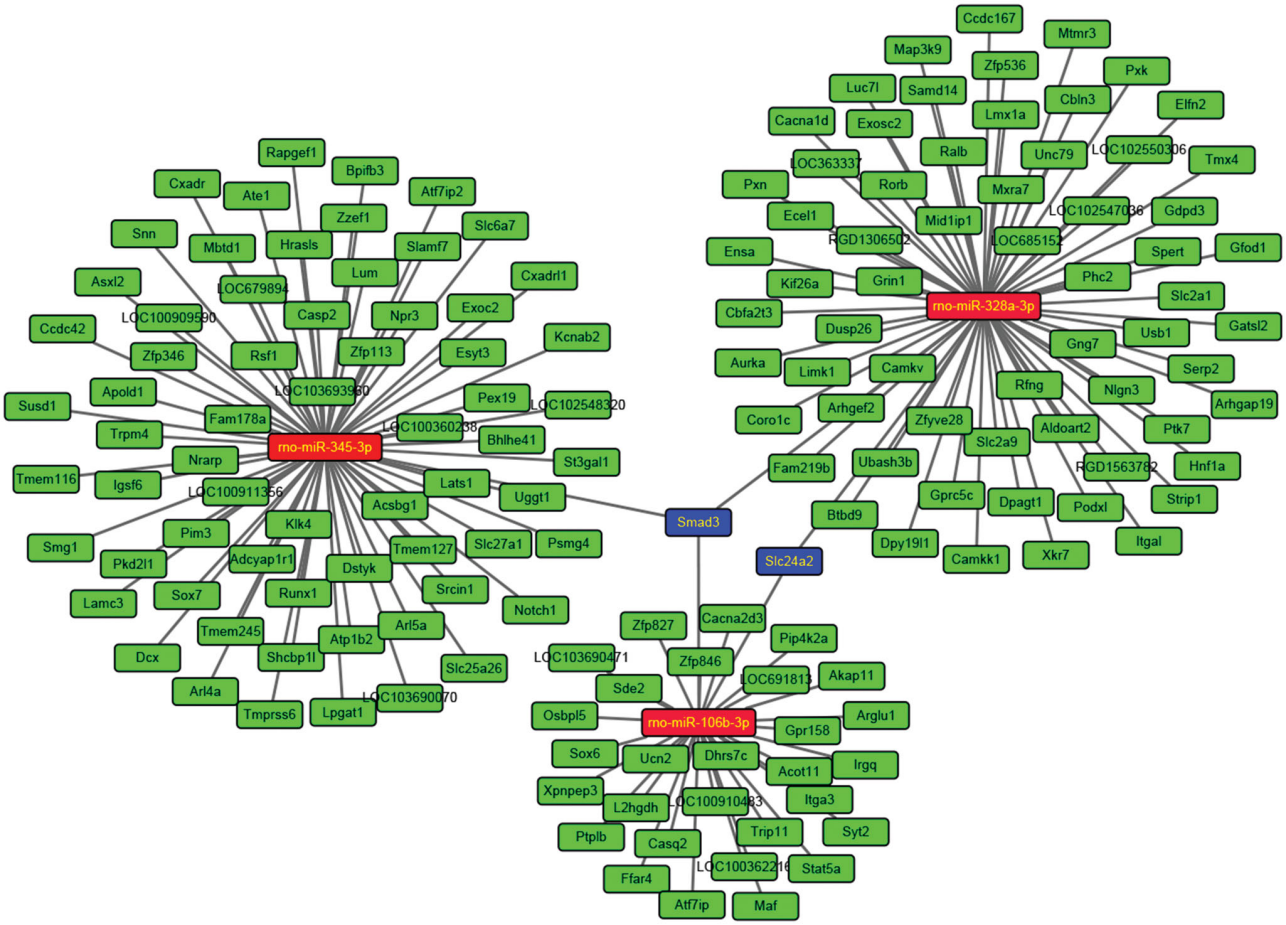
miRNA-mRNA network constructed from three differentially expressed miRNAs confirmed by qRT-PCR.

Thirty-three DEmiRs were detected in both two groups. Of these, 15 were upregulated and 18 were downregulated unlike in the CHF model. Volcano plot and hierarchical clustering analyses of the DEmiRs are shown in Figures 5(A,B), respectively. Computational DEmiRs targeting gene predictions suggested that Smad3 is a target of miR-345-3p, miR-328a-3p, and miR-106b-3p. These were downregulated in the CHF group unlike in the QL treatment group. RT-qPCR revealed that QL upregulated miR-345-3p, miR-328a-3p, and miR-106b-3p (Figure 6(A)). The miRNA–mRNA network for the three DEmiRs was created in Cytoscape (Figure 7).

### Smad3 is a target of miR-345-3p

As shown in Figure 6(B), co-transfection of the Smad3 reporter with miR-345-3p in HEK 293 cells resulted in a remarkable reduction in luciferase activity and indicated reduced levels of miR-328a-3p and miR-106b-3p. The MUTSmad3 reporter in combination with miR-345-3p did not alter the luciferase signal. Thus, miR-345-3p interacted directly and specifically with the 3′-UTR of Smad3. Hence, miR-345-3p directly regulates Smad3 mRNA in DOX-induced CHF.

## Discussion

Doxorubicin (DOX) is a practical anticancer drug with an extensive therapeutic range. However, its clinical use is severely limited by its side effects including cardiotoxicity, cardiac remodelling, and chronic heart failure (Lipshultz et al. 2012; Liu et al. 2016; Shabalala et al. 2017; Zhang et al. 2017; Chakraborti et al. 2018). DOX‐induced cardiotoxicity and CHF have been widely researched. However, the molecular mechanisms behind these adverse reactions have not been elucidated. Moreover, few clinically available drugs are effective against DOX‐induced cardiotoxicity. Qiliqiangxin (QL formula) is an extract comprising 11 different herbs. It has demonstrated positive inotropic, positive chronotropic, vasodilatory, anti-inflammatory, anti-apoptotic, myocardial antifibrotic, and diuretic efficacy in cardiac ischaemia and heart failure (Zou et al. 2012; Tang and Huang 2013). However, it is unclear whether and how QL enhances cardiac function and reduces myocardial remodelling in DOX-induced CHF. In this study, we explored the defensive and protective functions of QL in rats with DOX-induced CHF. Our data showed that whereas the DOX treatment caused heart failure, QL was cardioprotective in DOX-induced CHF rats. We found that QL inhibited apoptosis and the inflammatory response, promoted angiogenesis, and reduced myocardial fibrosis by inhibiting collagen production. Possible modes of action for QL are TGF-β1/Smad3 downregulation and TGF-β3/Smad7 upregulation. It was determined that Smad3 is a target gene for miR-345-3p and that QL may inhibit Smad3 by upregulating miR-345-3p. Overall, these results demonstrated that QL alleviates DOX-induced apoptosis, myocardial fibrosis, cardiac remodelling, and inflammatory response in CHF rats.

In the present study, 15 mg/kg DOX effectively induced CHF in rats. The hemodynamic parameters and echocardiography data showed that + dp/dtmax, −dp/dtmax, LVSP, EF, and FS were significantly lower and LVEDP, LVIDd, and LVIDs were significantly higher in the CHF model than the control. However, all of these parametric changes were reversed by QL. These results provided evidence that QL could improve myocardial function in DOX-induced CHF.

Angiogenesis is a vital repair process in myocardial ischaemia. The induction of angiogenesis should attenuate ventricular dysfunction and remodelling in DOX-induced CHF (Nessa et al. 2009). In addition, the Bax/Bcl-2 ratio affects sensitivity to mitochondrial apoptosis. Bax upregulation and Bcl-2 downregulation may be correlated with apoptosis in myocardial infarction (Misao et al. 1996). Our results showed that QL protects the heart against DOX cardiotoxicity by reversing Bax/Bcl-2 upregulation. Earlier studies reported that proinflammatory cytokines cause IκB and NF-κB phosphorylation and regression which, in turns, induce the transcription of inflammatory factors such as MMPs and TIMPs (Deten et al. 2002; Xie et al. 2004; Nam 2006; Timmers et al. 2009; Christia and Frangogiannis 2013). Here, MMP-2 and TIMP-2 levels were markedly upregulated in the cardiac model compared to those in the control. Nevertheless, QL inhibited MMP-2 and TIMP-2 overexpression. Thus, QL might attenuate DOX-induced cardiac inflammation and apoptosis in rats.

Cardiac extracellular matrix remodelling is an important mechanism of left ventricular dysfunction and adverse outcome after DOX treatment (Liu et al. 2016; Shabalala et al. 2017; Zhang et al. 2017; Chakraborti et al. 2018). Endogenous TGF-β plays a crucial role in the nosogenesis of myocardial remodelling and fibrosis. TGF-β modulates fibroblast phenotype and function (Mauviel 2005). The TGF-β superfamily comprises three structurally similar subtypes, namely, TGF-β1, TGF-β2, and TGF-β3. These are encoded by three distinct genes. TGF-β1 is the most prevalent subtype and is almost ubiquitous (Li and Brooks 1997). Several studies indicated that TGF-β1 promoted myofibroblast transdifferentiation via Smad3 signalling (Rosenkranz 2004; Bujak et al. 2007). The TGF-β1/Smad3 signal pathway may be a major driver of cardiac fibrosis. Smad7 competes with Smad3 and inhibits its phosphorylation (Wang et al. 2013). TGF-β1 upregulation and Smad7 downregulation are observed in myocardial remodelling. In contrast, Smad7 overexpression inhibits fibrotic responses by antagonising the TGF-β1/Smad3 signalling pathway (Wei et al. 2013; He et al. 2016). There is growing evidence that TGF-β is implicated in cardiac damage, repair, and remodelling. However, TGF-β1, TGF-β2, and TGF-β3 have unique mechanisms of modulating cardiac hypertrophy and fibrosis. In rat models of myocardial infarction, TGF-β1 and TGF-β2 are upregulated soon after MI whereas TGF-β3 is upregulated much later, primarily in the infarct section (Deten et al. 2001). In a model of pressure-induced hypertrophy, however, TGF-β3 is apparently antagonistic to TGF-β1. The latter is upregulated while the former is downregulated. Further, they are differentially distributed at the subcellular level during the development of left ventricular hypertrophy in rats (Li and Brooks 1997). In HSCs, TGF-β3 also has been found to significantly increase the mRNA expression levels of Smad6 and Smad7 but has no apparent effect on the mRNA levels of Smad3 and Smad4 (Deng et al. 2017). In the present study, we found that TGF-β1 and Smad3 were upregulated and TGF-β3 and Smad7 were downregulated in the CHF group relative to the control group. These findings did not necessarily prove a causal relationship between TGF-β3/Smad and collagens. Nevertheless, we proposed that TGF-β3/Smad7 attenuates myocardial remodelling and fibrosis in DOX-induced CHF, which merits further investigation. TGF-β3 could be a new therapeutic target in myocardial remodelling.

There is growing evidence that miRNAs participate in the onset and development of cardiac fibrosis (Verjans et al. 2018; Zhang et al. 2018). Here, we used miRNA sequencing to filter out the miRNAs related to TGF-β1/Smad3 signalling in DOX-induced CHF. Computational DEmiR target gene predictions disclosed that Smad3 was a target of miR-345-3p, miR-328a-3p, and miR-106b-3p. RT-qPCR revealed that miR-345-3p, miR-328a-3p, and miR-106b-3p were downregulated in CHF but upregulated in response to QL treatment. Moreover, luciferase reporter assays confirmed that miR-345-3p was directly implicated in the modulation of Smad3 mRNA in DOX-induced CHF. In summary, our results suggested that QL attenuated myocardial remodelling and fibrosis by inhibiting the TGF-β1/Smad3 signalling pathway, promoting the TGF-β3/Smad7 signalling pathway, and possibly inhibiting Smad3 by upregulating miR-345-3p. Thus, QL may serve as a new clinical therapeutic management strategy for cardiac fibrosis associated with DOX-induced CHF.

## Conclusions

In the present study, the QL treatment showed a strong cardioprotective effect in a DOX-induced CHF rat model. QL inhibited cardiomyocyte apoptosis, inflammation, and fibrosis and promoted neovascularization. Our findings implied that QL may exert its antifibrotic effect by inhibiting the TGF-β1/Smad3 signalling pathway and promoting the TGF-β3/Smad7 signalling pathway. Moreover, to the best of our knowledge, this was the first investigation to report the myocardial antifibrotic effect of the TGF-β3/Smad7 pathway. However, this discovery requires subsequent empirical validation. QL might also treat DOX-induced CHF by modulating specific miRNAs. The results of this research lay the foundation for further exploration into the protective effects and potential molecular mechanisms of QL in DOX-induced CHF.

## Data Availability

All data generated or analyzed during this study are included in this published article.
